# Optimizing diagnostic networks to increase patient access to TB diagnostic services: Development of the diagnostic network optimization (DNO) approach and learnings from its application in Kenya, India and the Philippines

**DOI:** 10.1371/journal.pone.0279677

**Published:** 2023-11-30

**Authors:** Heidi Albert, Sidharth Rupani, Enos Masini, Jeremiah Ogoro, Maureen Kamene, Donna Geocaniga-Gaviola, Eddie Sistoso, Celina Garfin, Sarabjit Chadha, Nishant Kumar, Kekeletso Kao, Zachary Katz

**Affiliations:** 1 FIND South Africa, Cape Town, South Africa; 2 LLamasoft, Inc., Ann Arbor, Michigan, United States of America; 3 World Health Organization, Nairobi, Kenya; 4 National Tuberculosis Leprosy and Lung Disease Programme, Nairobi, Kenya; 5 National TB Control Program, Department of Health, Manila, Philippines; 6 National Tuberculosis Reference Laboratory, Metro Manila, Filinvest, Philippines; 7 FIND, New Delhi, India; 8 Central TB Division, Ministry of Health and Family Welfare, Government of India, New Delhi, India; 9 FIND, Campus Biotech, Geneva, Switzerland; Stop TB Partnership, UNOPS, SWITZERLAND

## Abstract

Diagnostic network optimization (DNO) is an analytical approach that enables use of available country data to inform evidence-based decision-making to optimize access to diagnostic services. A DNO methodology was developed using available data sources and a commercial supply chain optimization software. In collaboration with Ministries of Health and partners, the approach was applied in Kenya, India and the Philippines to map TB diagnostic networks, identify misalignments, and determine optimal network design to increase patient access to TB diagnostic services and improve device utilization. The DNO analysis was successfully applied to evaluate and inform TB diagnostic services in Kenya, India and the Philippines as part of national strategic planning for TB. The analysis was tailored to each country’s specific objectives and allowed evaluation of factors such as the number and placement of different TB diagnostics, design of sample referral networks and integration of early infant diagnosis for HIV at national and sub-national levels and across public and private sectors. Our work demonstrates the value of DNO as an innovative approach to analysing and modelling diagnostic networks, particularly suited for use in low-resource settings, as an open-access approach that can be applied to optimize networks for any disease.

## Introduction

Expanding access to rapid diagnostics for the detection of tuberculosis (TB) and drug susceptibility testing (DST) is critical to reaching national and global TB control targets. The End TB Strategy [1] calls for the rapid diagnosis of TB and universal DST. To reach this goal, countries will need to establish a patient-centred and quality-assured diagnostic network equipped with rapid diagnostics recommended by the World Health Organization [2]. Access to diagnostic services at the point in the health system where patients first seek care has been identified as a key gap in the patient cascade for TB, despite significant investments in laboratory systems strengthening in recent years [3, 4]. Improving patient access while optimizing the utilization of existing diagnostic capacity is crucial to an efficient and cost-effective system. However, the optimal network design depends on a multitude of factors, including the specific epidemiology, geography, demography and health system of the setting, which can vary across countries and sub-nationally. Until recently, such decisions have been largely taken without systematic examination of trade-offs to design fully optimized diagnostic networks in an evidence-based manner.

Diagnostic network optimization (DNO) analysis is an innovative approach that uses data to enable evaluation and optimization of network configuration by balancing the need to increase patient access to services with cost efficiency and feasibility of implementation in resource-constrained settings [5–7]. Network optimization and strategic supply chain management using specialized software is widely used in the commercial sector to optimize resources. DNO is disease-agnostic and test agnostic, and similar modelling approaches have been used to inform the placement of TB diagnostics in Tanzania [8] and CD4 testing facilities in South Africa [9, 10].

This article describes the development of a comprehensive DNO methodology to strengthen diagnostic networks and how this approach has been applied inform national strategic planning and investment decisions for TB across a variety of different settings in Kenya, India and the Philippines.

## Methods

### DNO analysis scope

The DNO analysis for TB was designed to support Ministries of Health with identifying gaps and misalignments in diagnostic service delivery that can be addressed through laboratory strengthening interventions. Ethics approval was not required for this study as it did not involve the use of human subjects.

The DNO analysis aims to achieve the following high-level objectives:

1. Identify gaps and misalignments between diagnostic capacity and testing demand in the current (baseline) diagnostic network.
2. Test out potential optimization scenarios to address gaps and misalignments and recommend optimized network designs.
3. Project future demand for diagnostic testing and assess optimal network designs to meet future needs.
4. Model the placement of diagnostic tools (either already procured or planned) within the laboratory network and the estimated impact on patient access and network efficiency.

The analysis enables a sub-national differentiated modelling approach to diagnostic network strengthening based on regional epidemiological, demographic and geographical considerations. The specific scope of the analysis is adaptable and can be determined by the respective Ministries of Health and key stakeholders.

### DNO model structure

**Fig 1:**
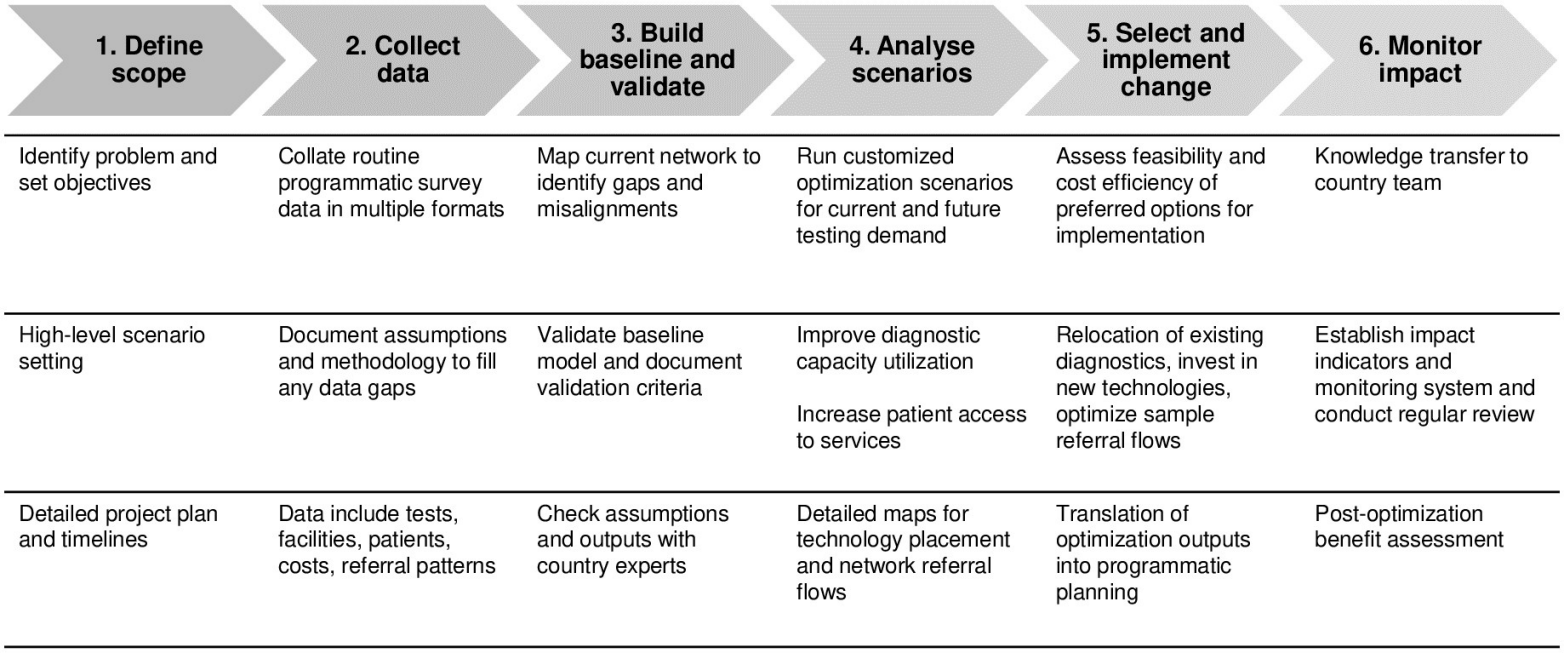
Diagrammatic representation of steps in diagnostic network optimization. This figure outlines the six key steps during diagnostic network optimization and main activities under each step.

The DNO model was constructed using commercial supply chain management design software, Supply Chain Guru^®^ 8.4 (SCG), together with a data management tool used for collating multiple data sources, DataGuru^®^ (Coupa Software Inc., San Mateo, CA, USA [LLamasoft, Inc, Ann Arbor, MI at the time of the project]). Fig 1 outlines the high-level steps involved in the DNO process. The network structure requires five main elements to build a model: Products, Sites, Demand, Sourcing Policies and Transportation Policies, as described below [11]. An overview of detailed inputs required for the five elements can be found in S1 and S2 Tables in S1 File. In addition to the basic data elements, constraints may be included relating to the above elements, such as constraints in selection of sites and referral of samples. For example, candidate locations for new devices are restricted to level 2 health facilities, sample referral across administrative boundaries are not considered.

#### Products

Diagnostic tests being conducted are defined as “Products” within the SCG software. A unique product name is given to each diagnostic test.

#### Sites

Sites include health facilities that order diagnostic tests (referring facilities) and facilities with laboratories and/or testing sites that conduct tests (referral facilities). Some sites may include both referring and referral capacity, e.g. a health facility that conducts smear microscopy but refers specimens to a higher-level facility for Xpert MTB/RIF or culture testing. Sites were linked for referral of samples for testing via Sourcing Policies and Transportation Policies.

#### Demand

Product demand denotes the diagnostic test orders requested within the network and was determined for WHO-recommended tests included in the national diagnostic algorithm. Testing data are generally entered as annual volume but may be entered as monthly or more frequent intervals, depending on data availability. Monthly or more frequent intervals are preferred where average testing volumes vary considerably over time (e.g. significant seasonal variations in testing volumes). Alternatively, an annual testing volume may be used with an adjustment factor which allows for a surge volume as a percentage of the average testing volume (e.g. surge factor of 1.3 means that the maximum volume is 1.3 times larger than the average volume).

Demand for testing was estimated at three levels: (a) baseline or current state demand, (b) intermediate demand, and (c) total demand. Baseline demand for testing was assumed to be equivalent to the actual testing conducted in the baseline year, based on reported laboratory testing data. Total demand was considered as the estimated volume of testing required to meet national case detection targets at the end of a defined period (usually the end of the current national strategic plan period). An intermediate demand estimate was made at an interim time point between baseline and total demand. Estimation of future testing demand was conducted using one of three approaches (summarized below with further information in the Supplementary materials):

- Composite diagnostic algorithmic framework: in this approach, a composite framework is constructed to illustrate all steps that patients may follow in the diagnostic pathway, from screening though to treatment monitoring, to estimate the number of tests required to reach TB case notification targets, as shown in S1 Fig in S1 File.
- Proportional growth demand: this method assumes that growth in demand occurs evenly across regions in a country compared with the baseline distribution of testing volumes and relies on calculating the proportional contribution of each sub-national region to the overall number of tests in the baseline year and applying the calculated regional proportion to the national estimated number of tests for the future year of analysis (illustration in S2 Fig in S1 File).
- Differential growth demand: this method applies the composite algorithmic framework at a regional level using disaggregated regional population data; national targets are applied for the proportions of people screened for TB and future testing demand is computed for each region using outputs from the algorithmic workflow. This results in a differential growth rate of testing demand in different regions compared with baseline, with testing demand being distributed differently across regions in the future compared with baseline year (illustration in S2 Fig in S1 File).

#### Sourcing and transportation policies

Sourcing policies define the linkages or relationships within the network model between referring health facilities that issue diagnostic test orders and the referral testing sites that perform the testing. Transportation policies define the actual route and mechanism by which samples travel from referring facilities to testing sites. Each sourcing policy requires a corresponding transportation policy.

Different sample referral and transportation systems are usually employed at different levels within the network. For example, motorcycle riders may be used at lower levels of the health system, such as between primary healthcare facilities and district hospitals, whereas courier services using motor vehicles may be used to transport samples to regional or national-level laboratories. Public transport may be utilized in certain areas. For estimating distances between primary healthcare centres and district health facilities, actual distances were used where available for existing transportation routes. Where actual distances between facilities and testing sites were not available, a distance adjustment factor was computed based on actual distance compared with straight-line distances on the map for known transport lanes in the same district or region and applied to routes with missing data. For estimating unknown actual distances, the straight-line distance was multiplied by the average transport adjustment factor. Each transportation mode (e.g. motorbike, car, bus, courier service, human carrier etc.) is represented as a transportation policy.

### Data sources and analysis

Data sources used to populate the model are shown in S3 Table in S1 File. These files were reviewed for completeness, plausibility and internal consistency of inputs, and were confirmed with stakeholders, before being merged and formatted into the SCG software’s model database schema. Data were compiled from various sources, most commonly in Microsoft Excel format.

Once data were inputted, scenarios (see Table 1) were created to test out various future state configurations for the network. This allows investigation of factors such as how changes in demand for testing (for one or more tests) affect optimal network design, how to improve access and increase utilization of current diagnostic capacity, different options for sample referral system design, and how to optimally incorporate future diagnostic devices. The resulting ‘optimized’ solution is the lowest cost solution that meets all constraints imposed upon the network model.

**Table 1:** Descriptions of scenarios and assumptions used in diagnostic network optimization.

Country *Time frame for analysis*	Key objectives	Capacity	Turnaround times for samples	Cross country sample referral	Notes
**Kenya *2017–2023***	Map current network Calculate future demand Assess utilization and location of GeneXpert instruments, EID integration and culture, DST and LPA Model optimal network design defined by NTP and partners, including new product and/or service investment	Baseline: current equipment and placement Intermediate demand: Free allocation of current equipment and free choice of any equipment type to any TB treatment centre (at least 1 instrument per county) Total demand: Best option from intermediate demand	2 days (easy to reach sites) 4 days (moderate sites) 7 days (hard to reach sites)	Within county referral Cross-county referral allowed Cross-county referral in south Kenya, within county in north Kenya	Pan-country analysis
**Philippines *2017–2022***	Map current network Calculate future demand Assess utilization and location of existing GeneXpert instruments, culture, DST and LPA Model optimal network design defined by NTP and partners, including new product and/or service investment	Baseline: Current GeneXpert device capacity and existing sample referral systems Optimized current demand: Current GeneXpert device capacity (300 instruments) and free allocation of 130 new GeneXpert4s to Level 2 and Level 3 hospitals nationwide Intermediate demand: current optimized design with any number and size of other GeneXpert instruments (GeneXpert 2, 4, 8, 16) to current DOTS locations Total demand: best option from intermediate demand scenario	6 days nationally, with 2 sample pick-ups per week (for GeneXpert)	Free sample referral allocation (initial analysis); 20 km maximum service distance from referring health facility to GeneXpert site (follow-up analysis)	Pan-country analysis Analysis of “Big 3 regions”*
**India *2018–2025***	Map current network Calculate future demand Assess utilization and location of existing CBNAAT instruments Model optimal network design for GeneXpert, Truenat instruments, sample referral systems, and culture, DST, LPA	Baseline: current equipment and placement			Pan-country analysis State-level analysis for Bihar, Assam and Karnataka

*Big 3 regions refer to the National Capital Region, Regions 3 and Region 4A

DOTS, Directly Observed Treatment Short course; DST, drug susceptibility testing; EID, early infant diagnosis of HIV; LPA, line probe assay; NTP, National Tuberculosis Program.

### Data visualization

Data visualization is a critical part of the process, both to identify gaps and misalignments in the current state (baseline) network, and to visualize outputs of different optimization scenarios to assess alternative options and feasibility for implementation. Data were visualized as maps prepared within the SCG software and as graphs and tables prepared by exporting data in csv or Microsoft Excel format. Maps included location of diagnostic services, colour-coded site icons to represent device utilization rates, and sample referral flows, at national and sub-national levels.

## Results

In collaboration with Ministries of Health, the DNO model was used to inform national strategic planning for TB in Kenya, the Philippines and India. Table 1 outlines the scope of the analysis covered in each country. The DNO approach was successfully adapted to each setting and provided insights to inform the strategic and cost-effective placement of TB diagnostics and design of optimal sample networks to improve patient access.

### Optimization scenario outputs

**Fig 2:**
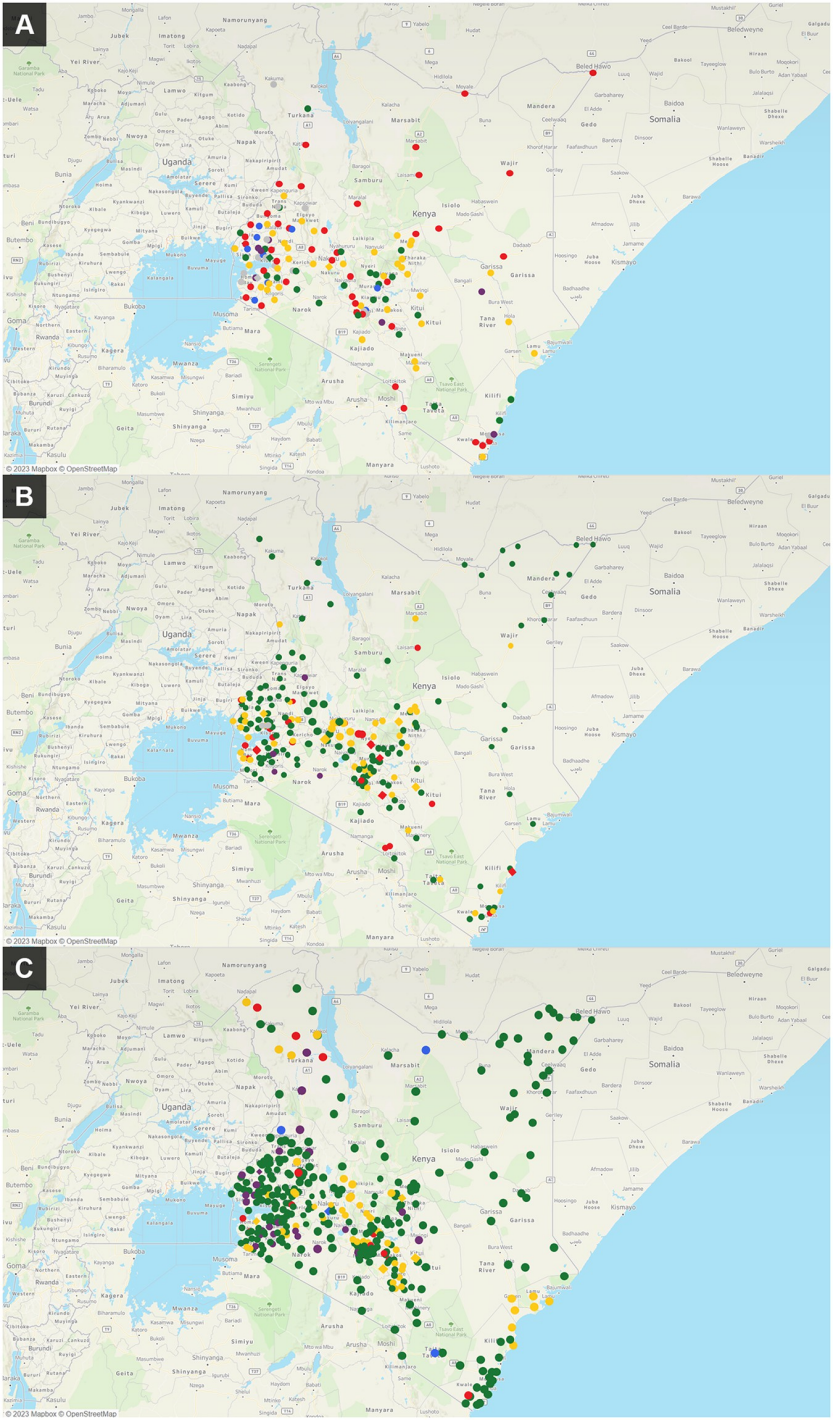
Comparison of the distribution and utilization of GeneXpert testing sites at (a) baseline, 2017, compared with predicted testing demand using National Strategic Plan case notification targets and differential growth estimate for (b) 2021 and (c) 2023 in Kenya. Optimized network design uses free allocation of testing demand. Optimized 2021 and 2023 networks use existing and potential new facilities. Base map and data from OpenStreetMap and OpenStreetMap Foundation.

**Fig 3:**
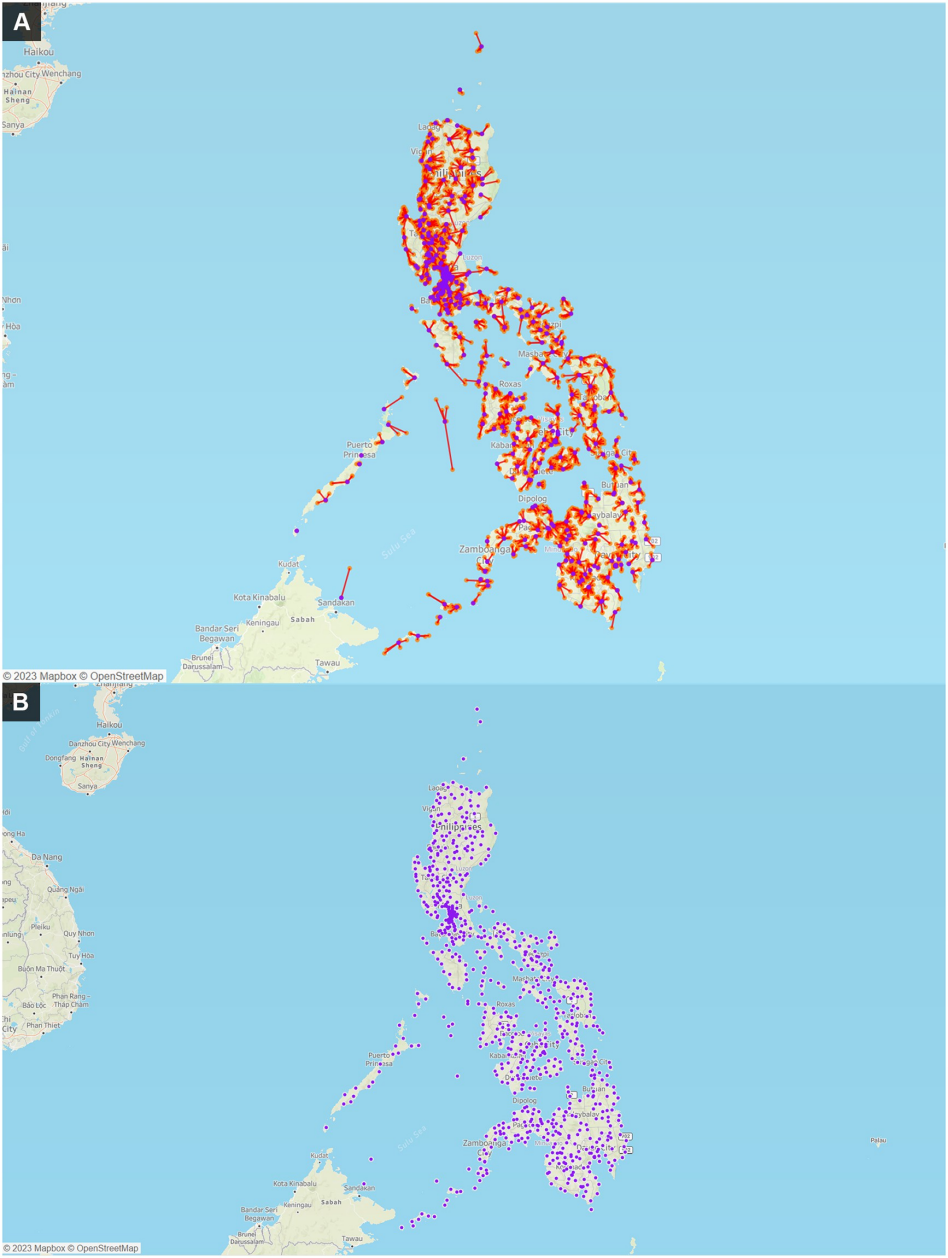
Representation of recommended device location and sample referral patterns based on the diagnostic network optimization model allocation of Xpert MTB/RIF testing demand in the Philippines without and with distance constraints in 2019. **A) Model allocation to meet 2019 demand levels, without distance constraints. B) Model allocation to meet 2019 demand levels, with 20 km service distance constraint**. Suggested locations of 859 Xpert machines across 796 sites to meet 2019 demand levels. Base map and data from OpenStreetMap and OpenStreetMap Foundation.

**Fig 4:**
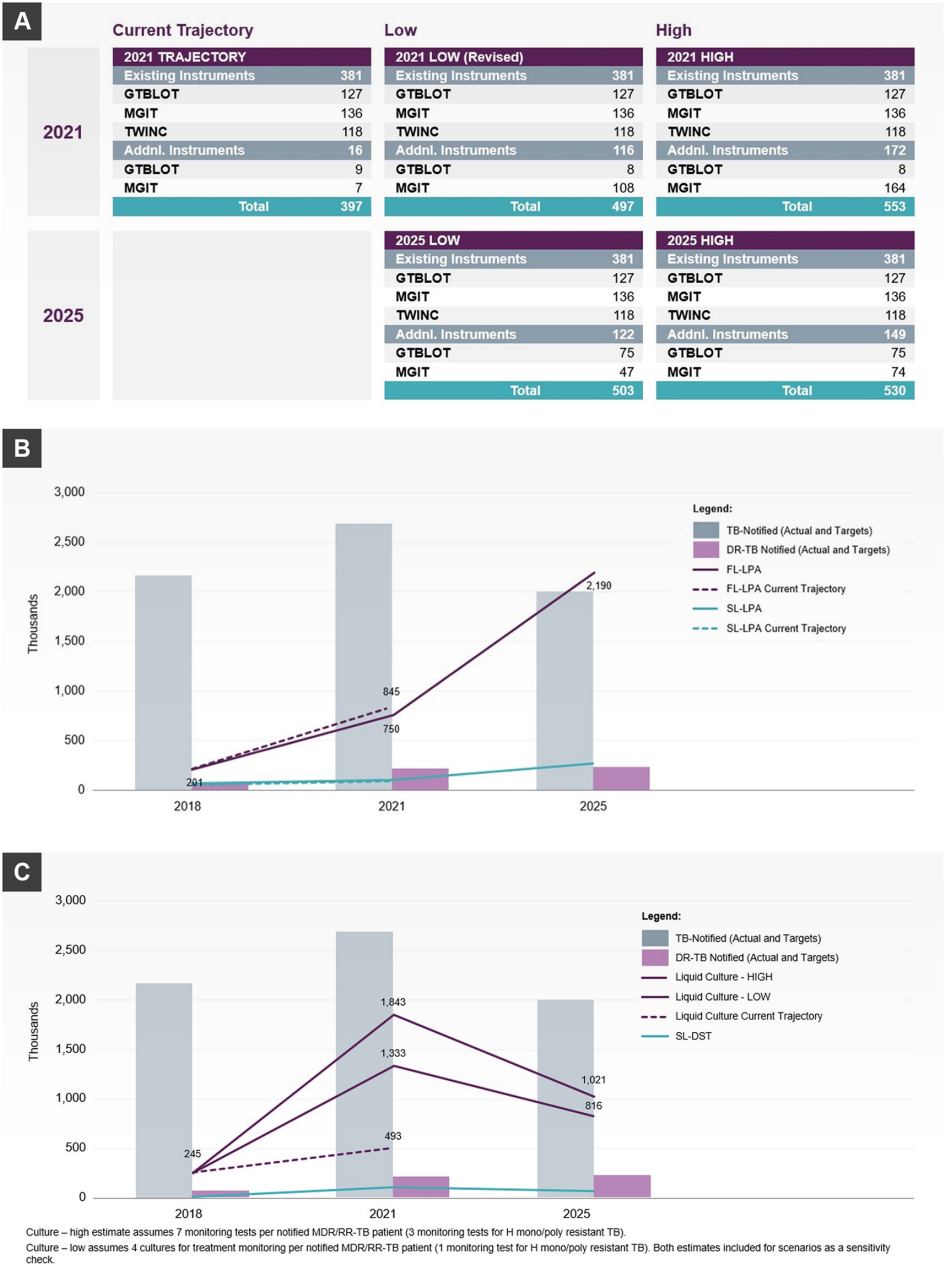
Selected DNO model outputs in India to meet future demand. **A) Number of additional instruments selected by the DNO model to meet future demand in India. B) Projected test demand and case notifications for LPA between 2018 and 2025 in India**. Demand for LPA is expected to increase ~4 times by 2021 in line with current trajectory and revised projections, with a large increase to 10 times current levels towards 2025 as NSP targets are followed. **C) Projected test demand and case notifications for culture tests between 2018 and 2025 in India**. Demand for culture tests is expected to increase ~5–8 times by 2021 to meet targets then substantially reduce towards 2025, as caseloads reduce. DST, drug susceptibility testing; DR-TB, drug-resistant tuberculosis; FL, first-line; LPA, line probe assay; MDR/RR-TB, multidrug-resistant/rifampicin-resistant tuberculosis; SL, second-line; TB, tuberculosis.

Outputs from key optimization scenarios across the countries are shown in Figs 2–4.

#### Kenya

In Kenya, the diagnostic network in 2017 had a total of 162 GeneXpert GXIV instruments and performed 275,000 Xpert MTB/RIF tests, significantly underutilizing available capacity (~466,500 tests per year). Demand projections for 2021 and 2023 estimated testing volumes would increase to 682,000 and 1.4 million tests, respectively. The model recommended that a total of 262 sites in 2021 and 450–500 sites in 2023 would be required to meet this demand, with many of the additional instruments recommended for placement in hard-to-reach counties to reduce turnaround time and improve access [12]. Model allocation of testing demand, based on minimizing volume-dependent referral distances between health facilities and testing sites, also resulted in overall improved utilization of instrument capacity (Fig 2). The DNO model was also applied to allocate demand for integrated testing of Xpert MTB/RIF and Xpert^®^ HIV-1 Qual (Cepheid, Sunnyvale, CA, USA) for early infant diagnosis (EID) of HIV infection on existing GeneXpert instruments (S3 Fig in S1 File).

#### Philippines

In the Philippines, DNO was applied to inform the optimal placement of GeneXpert devices across the different regions of the country, together with applying a maximum allowable distance constraint of 20 km from health facilities to the closest testing site. This resulted in 796 and 889 devices estimated for 2019 and 2022 projected testing demand levels, respectively. These estimates were significantly less than the approximately 1500 devices originally estimated by the country using simple projection computations prior to the analysis. Applying the model to sample referral systems also demonstrated how a referral pattern with free allocation can minimize overall sample transport distance. Fig 3 shows the optimized sample referral network based on the model’s allocation of Xpert devices to health facilities for 2019 demand levels without and with a 20 km distance constraint (shown in Fig 3a and 3b, respectively).

#### India

In India, the model identified that the current trajectory for culture and DST testing is insufficient to meet targets for testing. To meet total projected demand in 2025, the model recommends 47–74 Mycobacteria Growth Indicator Tube (MGIT) and 70–79 GT Blot instruments (for low and high demand estimates, respectively), spread over 10–12 states (Fig 4). Necessary testing capacity could be added through public sector equipment procurement for current facilities and/or establishment of new facilities, or through engagement of private sector facilities with existing MGIT capacity. While the model identified that line probe assay testing capacity is largely sufficient to meet current demand trajectory through 2021, from 2021 to 2025, capacity can be added through decentralized testing (e.g. using GeneXpert XDR instruments), more GT Blots, or a combination to balance cost and service/turnaround time requirements. The model was also applied to optimize and scale up high sensitivity molecular diagnostic tests in three states: Bihar, Assam and Karnataka. The state-wide application of DNO allowed for more granular evaluation of optimal diagnostic systems tailored for the local setting.

### Understanding key drivers of network design

The analysis undertaken in Kenya, the Philippines and India allowed for determination of the key drivers of optimal network design. One such driver is sample transport cost, which was found to be heavily dependent upon the frequency of transport. Consequently, countries may consider scenarios incorporating variable transport frequencies to determine the impact on overall cost efficiency balanced against ensuring adequate turnaround time for patient results. For example, in Kenya, groups of counties were categorized as “easy-to-reach”, “moderate” and “hard-to-reach” based on the size of the county, average distance between health facilities and testing sites, road infrastructure and intensity of partner support.

Another key driver of network design is the cost of setting up new testing facilities compared with establishing sample referral systems and increasing utilization of existing testing sites. It may be difficult to accurately estimate the total set up cost for new sites, and therefore a range of plausible costs should be applied to understand the impact on the model outputs. In the Philippines, the cost for setting up a new GeneXpert testing facility was estimated to be twice the annual operating cost of an existing facility.

Lastly, strengthening diagnostic systems in LMICs typically aims to increase access to diagnosis for underserved populations in the most cost-efficient manner. This may be achieved by placement of additional devices to enable on-site testing or via sample referral linkages. Where on-site testing is the preferred option, this must be balanced with the overall cost and the expected lower utilization of devices compared with more centralized network designs.

### Data visualization

A variety of visualization modalities were used including maps, graphs and tabular outputs, as illustrated in Figs 2 to 4. User-friendly visualizations enabled various stakeholders, most of whom did not have advanced data analysis expertise, to engage with the data and contribute towards evaluation of the current network status, validation of model assumptions, collaborative interpretation of the outputs, and development of customized interventions based on the local context. Maps showing the current status of the network (e.g. including site locations, testing capacity and utilization) were found to be a particularly useful format for data exploration by country stakeholders, and were used to identify or refine optimization scenarios.

### Implementation of recommendations

Recommendations from the DNO analyses in Kenya, India and the Philippines were used to inform network design and planning. In Kenya, recommendations from the DNO analysis were embedded in the diagnostics pillar of Kenya’s National Strategic Plan for tuberculosis, leprosy and lung health 2019–2023, which has been used to guide operational planning for TB since 2019 [12]. Following the National Strategic plan, additional DNO analysis was undertaken, including more detailed design of integrated sample referral systems, and updating of the national integrated sample referral guidelines (in collaboration with Centre for Health Systems Kenya, through USAID funded TB Accelerated Response and Care Programme II). This work built on the models and analysis described here, and included updated data on HIV integrated testing on GeneXpert devices and design of operational plans and budgets with the National TB Program (NTP), regional TB programme and laboratory officials from 15 counties.

In India, outputs were used to inform plans for GeneXpert placement and led to scaling up of DNO and route optimization analysis in 10 states (currently ongoing), which will be operationalized to improve access and turnaround time of molecular testing for TB. In the Philippines, site lists recommending optimal placement of GeneXpert devices were used to inform the number of devices procured and their placement in the network. The recommended designs for sample referral linkages were used in the operational planning of sample referral systems by the local implementing partner.

As data become available, models can be updated to reflect greater certainty around key inputs and assumptions. This iterative approach was undertaken in the Philippines to track progress against predictions and update optimized scenarios, providing relevant and timely updated recommendations that could be used to inform the implementation of national TB strategy.

## Discussion

The DNO approach enables use of available country data to inform rational evidence-based decision-making to optimize access to TB diagnostic services to support finding missing TB patients. The early and ongoing engagement of key country stakeholders ensures the model is pragmatic and tailored to the country context and facilitates teamwork in development of scenarios and validating model assumptions. The application of the tool in Kenya, the Philippines and India validated the DNO methodology and provided insights into the optimal placement and utilization of TB diagnostics. These insights were subsequently used to inform national strategic planning for TB and decisions regarding device procurement and placement. The outputs from this work also led to the development of an open access software (OptiDx) to enable expanded use of the approach in LMICs [13].

A strength of the DNO approach is that it can be tailored to suit national and local settings. The application of DNO in three different country settings also allowed identification of critical success factors and key learnings to inform future applications of the approach. Firstly, mapping the baseline diagnostic network early on in the process is important to allow adequate time for interrogation and validation of the country data, and engagement with country stakeholders. Planning sufficient time for the data collection phase is also essential, as collating data from various sources can be time-consuming, and verification and cleaning of data needs input from local data officers with knowledge of local data management systems. When considering the development of scenarios and interpreting their outputs, the feasibility of implementing the recommendations should be reviewed with local experts and implementation partners. For example, although modelling may find sample referral to be more cost-effective than placement of additional instruments at decentralized testing sites, implementation challenges such as insecurity, seasonal transportation challenges such as flooding, or other local factors may render such network designs as sub-optimal operationally. Longer working hours may be desirable at high-level facilities, e.g. regional hospitals, but infeasible at remote peripheral facilities with few staff. Similarly, a larger device footprint requires a more sophisticated system for management, and maintaining an uninterrupted supply chain for reagents may also prove burdensome compared with a smaller, less decentralized network.

Timing of the DNO is also critical to ensure it can be utilized to inform decision-making around strategic planning, funding and investment decisions. For example, decisions around instrument placement, particularly where this requires any infrastructure investment, should ideally be made several months in advance, to allow time for planning of site upgrades. Multiple partners or donors may be involved in supporting diagnostic networks, and therefore their planning and budgeting processes need to be taken into account and partners/donors need to be engaged early on in the DNO process to ensure the outputs are available in time for planning/funding cycles. Building the capacity of local stakeholders to conduct DNO is also important to allow teams to conduct exercises/updates independently as required.

Limitations of the DNO analysis should be taken into account when conducting and interpreting outputs of the analysis. A key challenge is that the analysis is heavily dependent on data availability, which may be limited or poor quality, particularly in LMICs. Use of expert opinion and sources of published or unpublished data to fill data gaps should be done with consideration of the potential impact of data uncertainties on the model outputs. Ongoing efforts to estimate sub-national disease burden [14, 15] can also be used to improve sub-national projections of future testing demand, which can be incorporated into the DNO model. In addition, sensitivity analyses are recommended to understand the effect of changes within the range of plausible inputs. It is important that the limitations of the model and uncertainty are clearly communicated to stakeholders when making recommendations. Value derived from DNO analysis may encourage countries to prioritize improving the quality of routine data required for the model in future years, to allow for more refined recommendations.

In summary, DNO is an emerging analytical approach that can be used to inform evidence-based strategic and operational planning to improve patient access to TB diagnostic services in a cost-efficient manner. The DNO method described here was successfully applied to develop customized and prioritized responses for strengthening TB diagnostic networks in Kenya, India and the Philippines. The DNO approach can be applied across a variety of diseases and settings and we recommend that DNO should form part of best practice in evidence-based programming to drive attainment of national goals.

## Supporting information

S1 File (DOCX)
