# Supplementary material for: Optimizing diagnostic networks to increase patient access to TB diagnostic services: Development of the diagnostic network optimization (DNO) approach and learnings from its application in Kenya, India and the Philippines
Source: PLoS One. 2023 Nov 30;18(11):e0279677. doi: 10.1371/journal.pone.0279677 (PMC10688908; doi:10.1371/journal.pone.0279677)
Supplement: S1 File — (DOCX) [file pone.0279677.s001.docx]

# Supporting information

**Table of contents:**

Supplementary Methods: page 2

Supplementary Tables: page 5

Supplementary Figures: page 11

Supplementary References: page 14

# S1 Methods

## Data sources

***Site data***

Availability of TB diagnostic and treatment services

Key sources of data on the availability and location of tuberculosis (TB) diagnostic and treatment services include National Health Service inventories and databases of health facilities providing TB services that are maintained by the National TB Program (NTP). Data on availability of smear microscopy, Xpert MTB/RIF testing, radiography and advanced tests including solid or liquid culture, molecular line probe assays to first- and second-line drugs, and drug susceptibility testing (DST) to first- and second-line drugs using phenotypic methods on solid or liquid media.

Health facility categorization

Data from different sources may use different categorizations when compiling data on health facilities. To adopt a standardized approach, we used the naming convention used in the patient pathway analysis (1), with each site being categorized according to (a) health sector, (b) health system level (1-5) and (c) health facility type (2). Categorization of health facilities was aligned with the country’s norms. Level 0 refers to basic, community-based care, commonly provided as extension services to facility-based care, such as community volunteer-provided treatment support for patients with TB. Level 1 (L1) facilities provide primary healthcare services, usually on an outpatient basis, and may include basic diagnostic services. Level 2 (L2) facilities, such as district hospital, provide primary healthcare plus additional diagnostic and treatment services and provide outpatient and inpatient care. Level 3 (L3) facilities, such as referral hospitals have more extensive inpatient capacity and have advanced diagnostic and treatment and provide specialized care.

***Demand data***

Estimating testing demand using a composite diagnostic algorithm framework

Testing demand at facility level is a key input required to construct the network model. Calculation of testing demand at facility level was performed by estimating the number of diagnostic tests in a given geographic area and then dividing this by the number of health facilities of different levels in that area. The geographic area selected was the lowest administrative area for which population, demographics and case notification data were available. In most cases this was the district or sub-district level. To allocate testing demand across health facilities within a district or sub-district, TB-specific care-seeking data from TB prevalence surveys were used. We used data from the patient pathway analysis for initial care seeking to allocate the proportion of patients within the sub-district administrative area to the health facilities within that area. Once the framework was constructed and populated for the baseline year, the outputs were validated against actual testing and NTP data on case notification at sub-regional level (county or region, depending on the lowest disaggregation of data available) using NTP data on case notification and laboratory tests reported that year.

Estimating current and future testing demand

Testing demand was determined for all World Health Organization-recommended tests included in the national diagnostic algorithm. For initial country analyses this has incorporated the following tests: sputum smear microscopy, Xpert MTB/RIF, TB culture, first (FL) and second-line (SL) phenotypic drug susceptibility testing (FL-DST, SL-DST respectively), line probe assay for first and second-line drugs (FL-LPA, SL-LPA respectively) and chest X-ray. Alternative World Health Organization-recommended tests may be incorporated into the algorithmic workflow where implemented as part of national algorithms in other countries. Testing demand for each test was determined at facility level, i.e. the facility at which the demand for testing originated, even where specimens were referred for testing elsewhere. This enabled the demand to be located at the site of initial patient care-seeking within the model and to generate network designs that respond to the need to improve patient access to services.

Estimation of future testing demand (intermediate and total demand) can be conducted using the three options described below. Selection of the approaches is dependent on the availability of disaggregated data and the required precision of the demand estimate. Alternative approaches to testing demand estimation may also be considered, but checks are recommended against the outputs from the below suggested methods as well as for internal consistency.

1. Composite diagnostic algorithmic framework,

To estimate the number of tests required to reach TB case notification targets, the composite diagnostic algorithm framework was constructed to illustrate all steps that patients may follow in the diagnostic pathway, from screening though to treatment monitoring (Supplementary Figure 1). This method is the most detailed estimation method and requires the greatest level of input data. The framework starts at the population level and allows estimation of the proportion of individuals that flow through each branch of the algorithm. Data sources that were used to construct the framework and populate the branches included National Prevalence Survey, NTP case notification data and annual reports, National Drug Resistance Surveys, laboratory data, and other sources (see Supplementary Table 1). This methodology allows for changes in input data at the regional level and can enable more precise modelling of the impact of interventions implemented at the regional level, or account for regional epidemiological or other variations.

1. Proportional growth

The proportional growth method is used to estimate future testing demand and assumes that growth in demand occurs evenly across regions in a country compared with the baseline distribution of testing volumes (illustrated in Supplementary Figure 2). Therefore, the highest contributing regions at baseline remain so in the future projection of testing volumes. This approach relies on calculating the proportional contribution of each sub-national region to the overall number of tests in the baseline year and applying the calculated regional proportion to the national estimated number of tests for the future year of analysis. This method assumes all regions have the same growth rate in the number of tests (the average national growth rate) and does not allow for a greater increase in testing among regions that may be under-performing at baseline, nor does it account for the impact of interventions that may be implemented in certain regions only.

1. Differential growth demand

The differential growth method applies the composite algorithmic framework at a regional level, using disaggregated regional population data (Supplementary Figure 2). National targets are applied for the proportions of people screened for TB, and for each region, the future testing demand is computed using outputs from the algorithmic workflow. This results in a differential growth rate of testing demand in different regions compared with baseline, with testing demand being distributed differently across regions in the future compared with baseline year. In cases of uncertainty or lack of data providing clear estimates of future sub-national demand for testing, both proportional and differential growth methods may be applied to provide a range of possible outputs that may be considered for discussion and validation with local experts.

***Costing data***

The cost per test was determined for each diagnostic test included in the network modelling and optimization exercise. For TB culture, the cost of positive and negative cultures was considered separately. The cost of conducting DST was determined based on the number of first- and second-line drugs tested in each country. Costs were broken down into the following components: (1) reagent costs, (2) equipment costs, (3) human resources and (4) quality assurance and training.

Reagent and equipment costs were taken from the Stop TB Partnership’s Global Drug Facility (GDF) Diagnostics Catalog (3) or local country pricing, depending on the procurement processes of the country. Costs not available from GDF were estimated using the Expand-TB Budget Application Tool (4). The cost of international and local shipment of reagents and equipment, together with any locally applicable surcharges or duties were included.

To allocate the cost of equipment per test, the following formula was applied:
*Equipment cost per test = Equipment purchase price / number of tests performed per year x equipment lifespan (yr)*

The maximum number of tests that could be performed per year was taken from GLI Practical Guide to TB Laboratory Strengthening.(5) The instrument lifespan was based on depreciation rates of similar equipment (General Depreciation Rates-IRD New Zealand).

Human resource cost per test was determined based on country staffing norms and salary rates, multiplied by the estimated time to conduct a test. The estimated time to conduct tests was obtained from published sources (5, 6). Estimated staff salaries were taken as the mid-range laboratory technician salary reported on a salary comparison website, and was the average monthly salary including housing, transport, and other benefits (7). Costs were adjusted to the average US Dollar amount for the year of the analysis (8).

## Costs for quality assurance and training included estimates for proficiency testing, maintenance and calibration, training and on-site supervision visits. It was assumed that proficiency testing and on-site supervision occurred once per year and on-site supervision included all diagnostic tests conducted at a given site (i.e. one visit per site per year). The cost of proficiency testing was taken from GDF Diagnostic Catalog or test manufacturers. Cost of training and on-site supervision was determined in consultation with local laboratory experts and based on experience from the FIND team. Maintenance and calibration costs were obtained from the manufacturer or local authorized service providers. For set up of new sites in future scenarios, the cost of set up was estimated to be twice the annual cost of quality assurance and training. An additional cost was added in year 1 to cover infrastructure modifications and minor renovations for newly established GeneXpert labs and culture/DST/LPA laboratories based on country experience.

# Supplementary Tables

**S1 Table. Detailed inputs for Products, Sites and Demand Tables required for diagnostic network model construction using commercial supply chain management software**

| **Product Table** | |
| --- | --- |
| **Product name** | Unique name of diagnostic test (may be further disaggregated by including specimen type or patient classification where this impacts on network design, e.g. viral load testing for routine monitoring or viral load testing for pregnant and breastfeeding women may be defined as discrete products). |
| **Sites Table** | |
| **Facility Name** | Full facility name |
| **Facility ID** | Unique facility identifier (if available) |
| **Country** | Country name |
| **Region/Province/County** | Region/province/county name |
| **Sub-district administrative area** | Name of lowest administrative area |
| **Health sector** | Public, formal private, informal private |
| **Health system level** | Level 1, 2, 3, 4, 5* |
| **Geo-location** | Site longitude and latitude |
| **Which of the following tests does the facility perform?** | Smear microscopy, light Smear microscopy, LED FM Smear microscopy, FM Xpert MTB/RIF Chest X-ray TB culture and identification - liquid medium TB culture and identification - solid medium Line probe assay (LPA) for first line drugs LPA for second line drugs Phenotypic drug susceptibility testing (DST) for first line drugs (liquid or solid media) Phenotypic DST for second line drugs (liquid or solid media) |
| **Which of the following services does the facility provide?** | TB treatment ART PMTCT EID |
| **Site type** | The site type can be categorized in one of three ways, based on the site’s role in the network model.  **Existing Facility**. A site that exists in the model structure by default.  **Potential Facility**. A site that does not exist in the model structure by default, but is tested during optimization for its potential contribution to the network.  **Customer.** A site that initiates demand for product (i.e. orders tests). |
| **Site Status** | The status of the site determines its inclusion of the site in any model optimization or simulation calculations.  **Include.** The site is included in optimization by default.  **Exclude.** The site is not included in optimization by default.  **Consider.** Network optimization recommends whether the site should be used to create an optimal network structure. |
| **Demand Table** | |
| **Customer** | The site that orders the test |
| **Product** | The test that is ordered |
| **Quantity** | The quantity of the test ordered by a customer, usually entered as annual or monthly quantity |

ART, anti-retroviral therapy; DST, drug susceptibility testing; EID, early infant diagnosis (for HIV); ID, identification; LED FM, Light-emitting diode-based fluorescence microscopy; LPA, line probe assay; PMTCT, prevention of mother to child transmission; TB, tuberculosis.

**S2 Table. Description and requirements for establishing Sourcing Policies and Transportation Policies for diagnostic network optimization analysis**

| **Sourcing Policies** | |
| --- | --- |
| **Source name** | Name of site or sites from which tests can be sourced - Can be restricted to 1 site (e.g. in current state), allow any sites, or allow specified groups of sites |
| **Site name** | Name of site requesting a test |
| **Product name** | Test name that is served by the Sourcing Policy |
| **Sourcing policy type** | **Single source** - Test can be sourced from one source facility only. The source facility is selected based on the optimization objective (usually minimum cost).  **Multiple sources (most inventory)** - tests are sourced from multiple source facilities. Selection of source facilities is based on feasibility and cost considerations. |
| **Sourcing policy allocation** | **Current state flow** - sourcing is as per current network structure and flows **Free allocation** - sites are able to source tests from any sites within the network with the requisite test menu and capacity **Allocation with restrictions** (e.g. sourcing only allowed within regional boundaries) |
| **Transportation Polices** | |
| **Source name** | Name of site or sites from which tests can be sourced - Can be restricted to 1 site (e.g. in current state), allow any sites, or allow specified groups of sites |
| **Site name** | Name of site requesting a test |
| **Transport mode** | Method of transportation between the referring and testing site. Common transport modes include motorbike, courier services, public transport, health facility vehicle, hand carriage |
| **Product name** | Transportation policies may apply to all tests or different transportation policies may be applied. If product name is not included in the policy, then all products can flow using the policy. |
| **Transportation policy type** | A variety of transportation policies are provided for in the software that reflect different types of transportation policies. Common examples include the following:  **Less than truckload (LTL)** – samples are shipped immediately, without taking into account shipment of other samples going to the same destination.  **Full truckload (FT)** – samples are bundled together for shipment, based on shipment weight or volume.  **Daily, Weekly or Periodic Shipments** (as per defined scheduled).  If detailed data are not available at the time of the analysis, LTL is preferred for initial analysis. More detailed sample referral system design may be conducted in a second phase of work. |

**S3 Table. Data requirements and suggested data sources for diagnostic network optimization analysis**

| **Variable** | **Data requirement** | **Description** | **Data sources** |
| --- | --- | --- | --- |
| **Health Facility** | | |  |
| Health Facility (locations) | Facility Master List (FML) | List of health facilities including:   - geolocations, - health sector (public, private informal, private formal) - facility level - urban/rural classification - TB services offered including diagnosis and treatment of DS-TB and DR-TB - Other services offered that may impact on TB services, e.g. ART, PMTCT, EID | District Health Information Software (DHIS) data and/or other health facility lists compiled by Ministries of Health |
| **Demand** | | | |
| Population (Catchment area of HF) | Health facility catchment area | Size of population served by each health facility | Census and/or electoral data together with assumptions on health seeking behaviour, e.g. from health service utilization surveys or Demographic and Health Surveys (DHS) |
| Estimated number of cases for each HF (from prevalence rate) | TB prevalence rate at each health facility | National TB prevalence data broken-down to facility level – last full year of data, by health facility, by quarter | NTP reports |
| Number of patients seeking care at a particular HF | All patients seeking services at a HF | Number of patients that seek services at each health facility preferably broken-down by quarter | Census and/or electoral data together with assumptions on health seeking behaviour, e.g. from health service utilization surveys or Demographic and Health Surveys (DHS) |
| Number of patients screened for TB at a particular HF | Patients screened for TB signs and symptoms at each HF | Of the number of patients seeking care at each health facility those that are screened for signs and symptoms at each health facility preferably broken-down by quarter | NTP may compile such data at business management unit (BMU) or district level. Such data are often not compiled and reported to national level |
| Number of presumptive TB patients at a particular HF | Presumptive TB patients at each HF | Of the patients that are screened for TB those identified as having TB signs and symptoms at each health facility preferably broken-down by quarter | NTP may compile such data at business management unit (BMU) or district level. Such data are often not compiled and reported to national level. May be estimated from other NTP data |
| Number of patients with access to diagnostics at a particular HF | Number of presumptive TB patients with a lab result | Number of presumptive TB patients that get a laboratory test to diagnose TB broken-down by diagnostic tool used   - Smear microscopy - Xpert MTB/RIF or Ultra - Culture and DST | NTP reports |
| Number of diagnosed TB cases at a particular HF | Number laboratory confirmed TB cases | Number of presumptive TB cases confirmed as having TB by   - Smear microscopy - Xpert MTB/RIF or Ultra - Culture and DST | NTP reports |
| Number of TB cases at a particular HF | Number of TB cases registered at a HF | Number of all TB cases (clinically diagnosed and lab confirmed) identified at each health facility preferably broken-down by quarter | NTP reports |
| Number of TB cases initiated on TB treatment at a particular HF | Number of TB cases put on treatment at a HF | Out of all the TB cases those that are put on treatment. | NTP reports |
| Number of patients presumptive DR-TB patients at a particular HF |  | Number of patients that meet the criteria for being classified as presumptive DR-TB. Criteria may include:   - Treatment failure - Relapse - Treatment after loss to follow up - Contact of DR-TB patient | NTP reports |
| Number of DR-TB patients diagnosed at a particular HF | Number laboratory confirmed DR-TB cases | Number of presumptive DR-TB cases confirmed as having DR-TB by   - Xpert MTB/RIF or Ultra - Culture and DST - LPA | NTP reports |
| Number of DR-TB cases initiated on TB treatment at a particular HF | Number of DR-TB cases put on treatment at a HF | Number of notified DR-TB cases initiated on treatment | NTP reports |
| **Diagnostic Network** | | | |
| Referral pattern | Design of referral network | Data on which facilities refer to which testing sites for all testing broken-down by technology used:   - Smear microscopy - Xpert MTB/RIF or Ultra - Culture and DST - LPA - Radiography | Data may be compiled at NTRL for culture/DST/LPA referrals. For smear and Xpert MTB/RIF this may be collated at district level or higher, depending on setting. Radiography referral patterns may not be documented or restricted to lower administrative levels only (e.g. district). Availability of data may vary between public and private sector |
| Which facilities have diagnostic services | All health facilities with an X-ray | List of health facilities with diagnostic services and quantity of instruments (capacity) available at each HF, broken down by technology used:   - Smear microscopy - Xpert MTB/RIF or Ultra - Culture and DST - LPA - Radiography | Data may be compiled at NTRL for culture/DST/LPA referrals. For smear and Xpert MTB/RIF this may be collated at district level or higher, depending on setting. Radiography data may be collated by NTP or research projects, or at lower administrative levels only (e.g. district). Availability of data may vary between public and private sector |
| **Costs** | | | |
| Transport costs | Cost of the sample referral systems | Costs of sample transportation across different levels of the network.   - Which sample transport mechanisms are used? - Are costs based on per sample or per journey? - Standard or variable cost per distance? - Is transport on demand or regular scheduled service? | Information may be available from sample transport service providers, including couriers, implementing partners or district health offices |
| Equipment – operating costs |  | Warranty/service and maintenance costs, cost of reagents and supplies. Includes:   - Shipment - Human resources - Infrastructure - Training - Quality assurance | NTP / NTRL / Manufacturer or authorized service providers |
| Equipment – capital costs |  | Purchase costs of instruments, including shipment, customs costs, etc. | NTP / NTRL / Manufacturer or authorized service providers |

HF, health facility; TB, tuberculosis; DR-TB, drug resistant tuberculosis; EID, early infant diagnosis (of HIV); NTP, National Tuberculosis Programme; NTRL, National Tuberculosis Reference Laboratory; PMTCT, prevention of mother to child transmission; DST, drug susceptibility test; LPA, line probe assay

**Supplementary Figures**

**S1 Fig: Diagrammatic representation of the Composite Diagnostic Algorithmic framework for estimation of diagnostic testing demand**


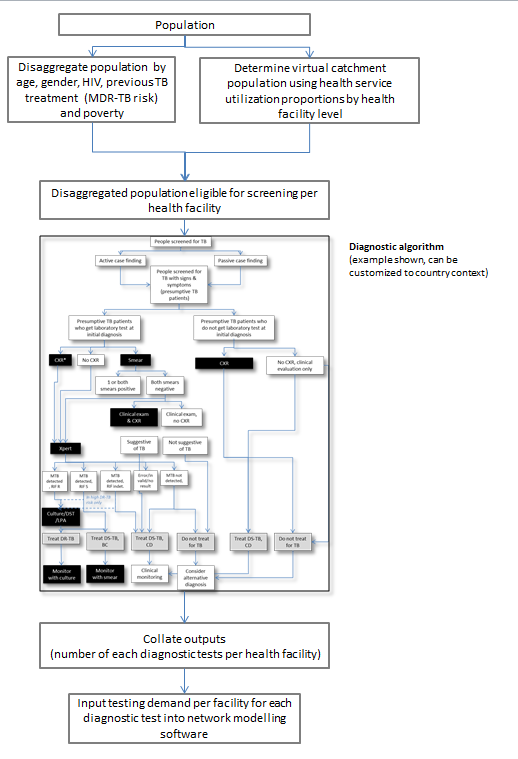


**S2 Fig. Illustrative example of testing demand estimation using Proportional Growth (PG) and Differential Growth (DG) estimation method**

An illustrative country example consisting of five regions. Baseline average 74% of estimated cases notified, varying percentage in different regions. Future number of TB cases notified calculated using either DG or PG method. DG method consists of applying target of 90% of estimated cases notified to all regions, while PG method applies target nationally with regions contributing to the national case finding based on proportional contribution of cases at baseline.

**
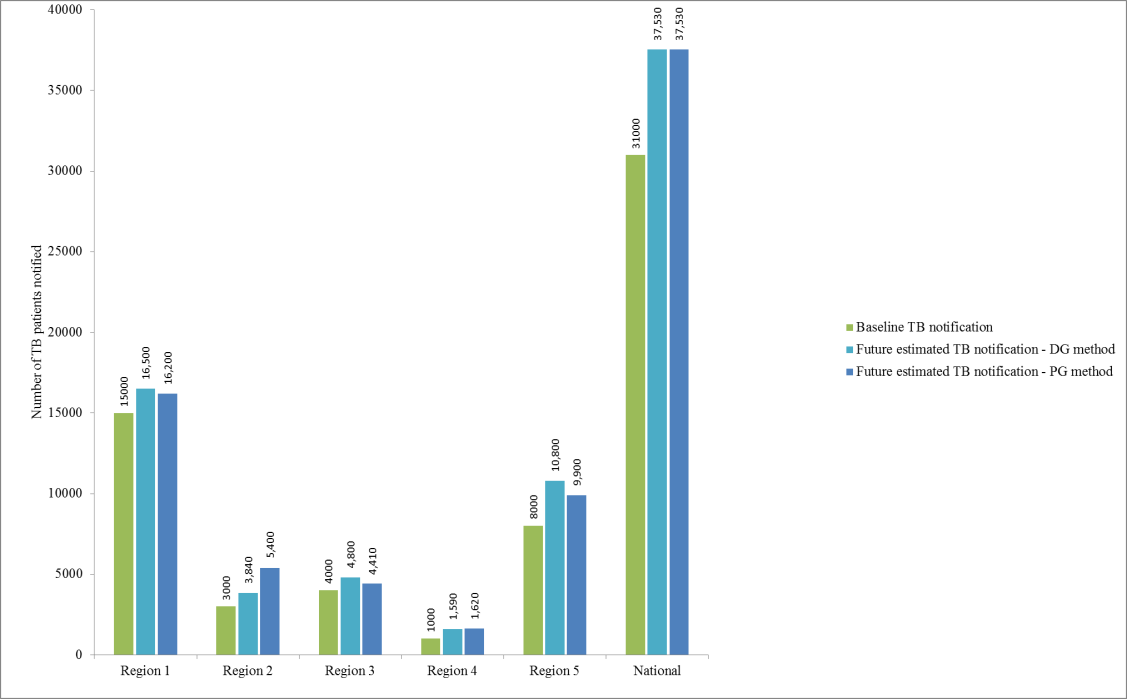
**

**S3 Fig. Allocating demand for integrated testing of Xpert MTB/RIF and Xpert^®^ HIV-1 Qual for early infant diagnosis (EID) of HIV infection on existing GeneXpert instruments at testing sites in Kenya.** In this example, the testing demand was allocated in parallel to TB and EID testing.


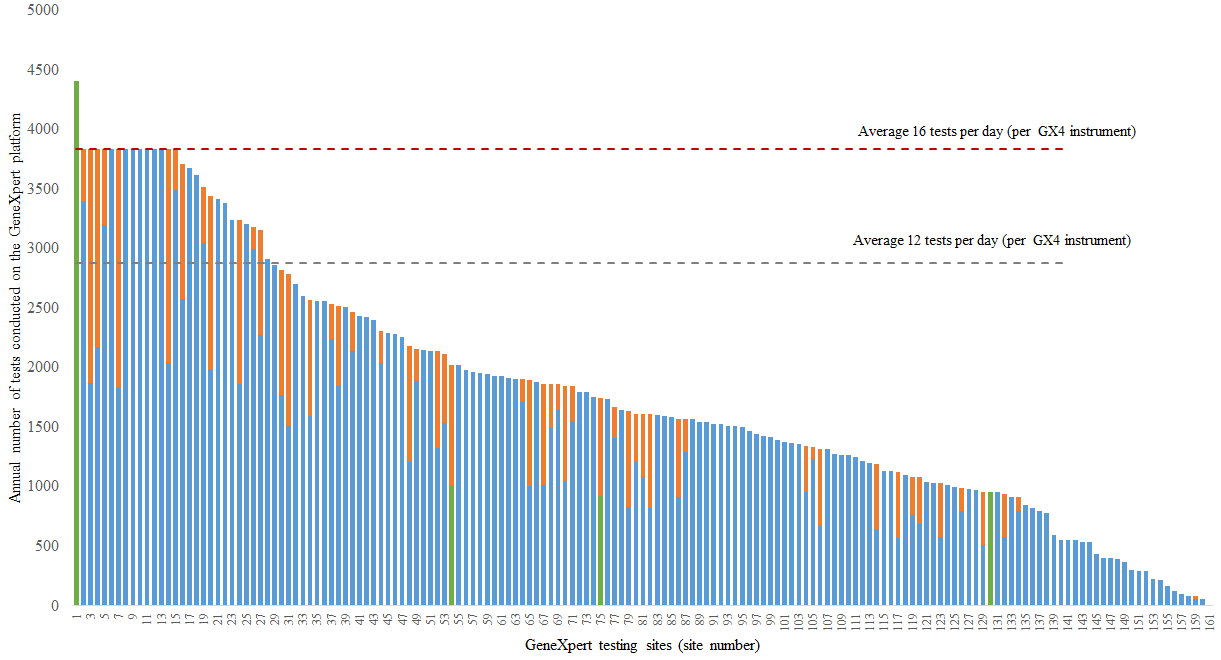


Blue: Xpert MTB/RIF testing; orange: EID testing. Green lines indicate sites with GeneXpert 16-module instruments. All sites with Xpert MTB/RIF testing in blue are equipped with GeneXpert 4-module (GX-4) instruments.

**Supplementary References**

1. Hanson CL, Osberg M, Brown J, Durham G, Chin DP. Conducting Patient-Pathway Analysis to Inform Programming of Tuberculosis Services: Methods. J Infect Dis. 2017;216(suppl_7):S679-S85.

2. Osberg M, Brown J, Hanson C, Seabrook D, Chaphiv M, G D. Patient Pathway Analysis : How-To Guide. 2017.

3. Stop TB Partnership - Global Drug Facility (GDF). GDF Diagnostics Catalog 2018 [Available from: <http://www.stoptb.org/assets/documents/gdf/drugsupply/GDFDiagnosticsCatalog.pdf>.

4. Expand-TB. Expand-TB Budget Application Tool 2015 [Available from: <https://www.finddx.org/wp-content/uploads/2016/04/Budgeting-Application-Tool-UserManual-English-16Oct15.pdf>.

5. Global Laboratory Initiative. GLI Practical Guide to TB Laboratory Strengthening 2017 [Available from: <http://stoptb.org/wg/gli/assets/documents/GLI_practical_guide.pdf>.

6. Mathys V, Roycroft E, Raftery P, Groenheit R, Folkvardsen DB, Homorodean D, et al. Time-and-motion tool for the assessment of working time in tuberculosis laboratories: a multicentre study. Int J Tuberc Lung Dis. 2018;22(4):444-51.

7. Salary Explorer. Salary and Cost of Living Comparison 2019 [Available from: <http://www.salaryexplorer.com/>.

8. Oanda. Currency Converter 2019 [Available from: <https://www1.oanda.com/currency/converter/>.
